# Supplementary material for: The causal relationship between the human gut microbiota and pyogenic arthritis: a Mendelian randomization study
Source: Front Cell Infect Microbiol. 2024 Nov 26;14:1452480. doi: 10.3389/fcimb.2024.1452480 (PMC11629706; doi:10.3389/fcimb.2024.1452480)
Supplement: Supplementary file 1 [file DataSheet1.zip › mendelian test/ebi-a-GCST90027556.csv_forest.pdf]

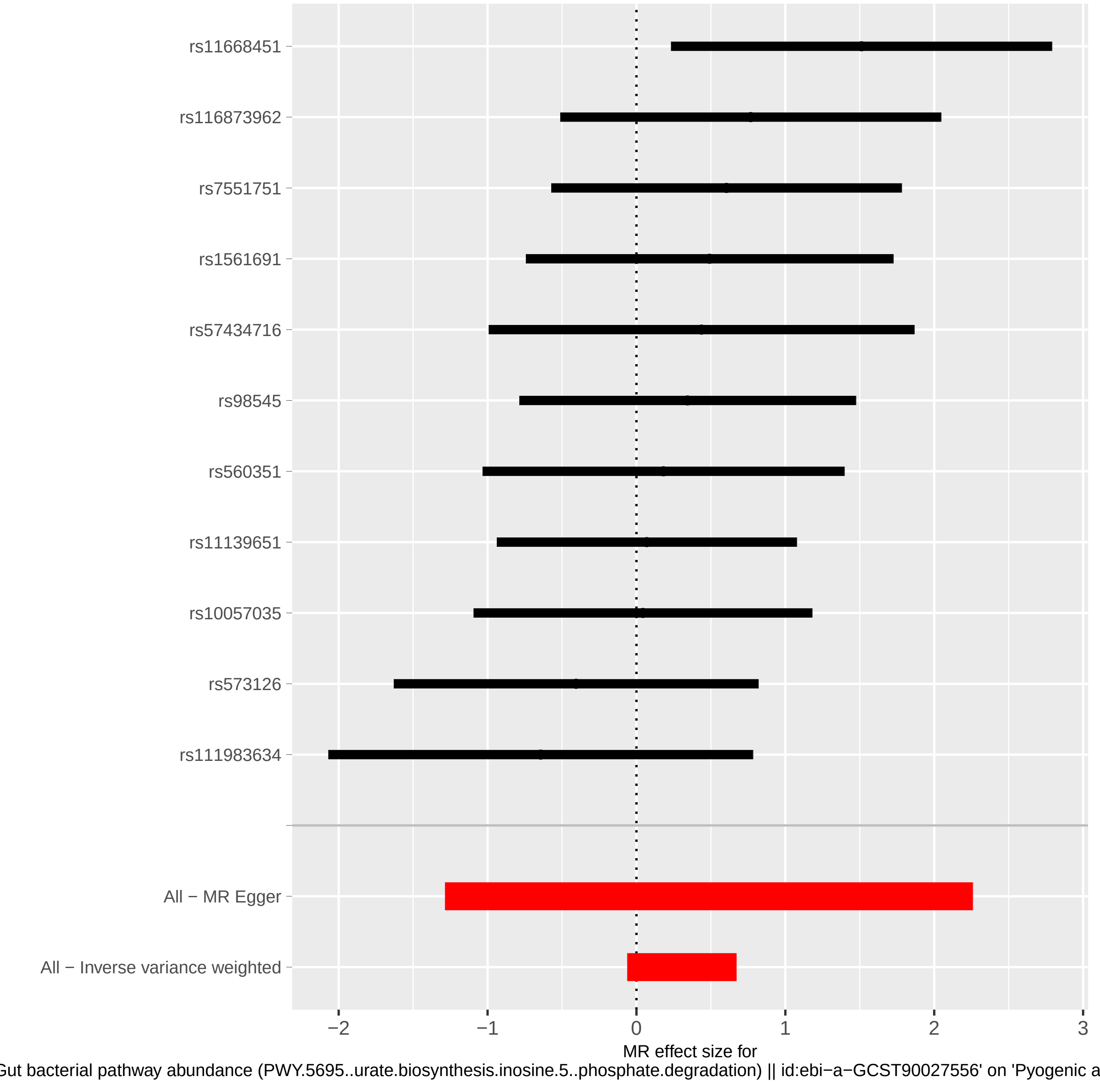
rs11668451
rs116873962
rs7551751
rs1561691
rs57434716
rs98545
rs560351
rs11139651
rs10057035
rs573126
rs111983634
All − MR Egger
All − Inverse variance weighted
−2
−1
0
1
2
3
MR effect size for
Gut bacterial pathway abundance (PWY.5695..urate.biosynthesis.inosine.5..phosphate.degradation) || id:ebi−a−GCST90027556' on 'Pyogenic a
